# Quantitative measures to assess the quality of cellular indexing of transcriptomes and epitopes by sequencing data

**DOI:** 10.3389/fbinf.2025.1630161

**Published:** 2025-09-18

**Authors:** Jie Sun, Robert Morrison, Soyeon Kim, Kairuo Yan, Hyun Jung Park

**Affiliations:** 1 Department of Human Genetics, School of Public Health, University of Pittsburgh, Pittsburgh, PA, United States; 2 Department of Medicine and Division of Hematology/Oncology, University of Pittsburgh, School of Medicine, Pittsburgh, PA, United States; 3 Department of Immunology, University of Pittsburgh, School of Medicine, Pittsburgh, PA, United States; 4 Department of Computational and Systems Biology, University of Pittsburgh Medical Center, Pittsburgh, PA, United States; 5 Division of Pulmonary Medicine, Department of Pediatrics, UPMC Children’s Hospital of Pittsburgh, University of Pittsburgh, Pittsburgh, PA, United States; 6 Department of Computer Science, Northeastern University, Boston, MA, United States

**Keywords:** CITE-Seq, quality control (QC), multi-omics integration, biomarker discovery, computational software

## Abstract

**Background:**

Cellular indexing of transcriptomes and epitopes by sequencing (CITE-Seq) is a powerful technique to simultaneously measure gene expression and cell surface protein abundances in individual cells. To obtain accurate and reliable biological findings from CITE-Seq data, it is critical to ensure rigorous quality control (QC). However, no public method has yet been developed for CITE-Seq QC.

**Results:**

In this study, we propose the first software package for multi-layered, systemic, and quantitative quality control (CITESeQC). Recognizing the multi-layered nature of CITE-Seq data, CITESeQC performs QC across gene expressions, surface proteins, and their interactions. It systemically evaluates all genes and protein markers assayed in the data and filters out some of them based on individual quality measures. Furthermore, for quantitative QC that enables objective and standardized analyses, CITESeQC quantifies cell type-specific expression of genes and surface proteins using Shannon entropy and correlation-based measures. Finally, to ensure broad applicability, CITESeQC guides users through a simple process that generates a complete markdown report with supporting figures and explanations, requiring minimal user intervention.

**Conclusion:**

By quantifying the quality of CITE-Seq data, CITESeQC enables precise characterization of gene expression within cell types and reliable classification of cell types using surface protein markers, thereby enhancing its value for clinical applications.

## Background

While traditional single-cell RNA-seq techniques assay only gene expression by capturing and sequencing RNA molecules, cellular indexing of transcriptomes and epitopes by sequencing (CITE-Seq) assays both RNA molecules and surface proteins of interest simultaneously by utilizing unique DNA-barcoded antibodies, also known as “antibody-derived tags (ADTs).” Since cell surface proteins serve as markers and communicators of a cell’s identity and function, CITE-Seq data enable the identification not only of cell-type specific gene expression patterns but also of cell types defined by specific surface proteins that may be used for further clinical applications. For example, although some immune cell types, such as γ/δ T cells (Zakeri et al., 2022), mucosal-associated invariant T cells (Li et al., 2023), innate lymphoid cells (ILCs) (Jacquelot et al., 2022), and neutrophils (Geh et al., 2022), have demonstrated significant clinical potential, single-cell RNA-seq data alone are often insufficient to detect them reliably. This limitation arises from the potentially low RNA content of lineage-defining transcripts (Stoeckius et al., 2017), the presence of high levels of RNase (Hao et al., 2021; Mazzurana et al., 2021; Scheyltjens et al., 2022), or the fact that mRNA expression patterns do not always correlate with protein expression (Stoeckius et al., 2017).

To ensure high-quality discoveries from CITE-Seq data, the first critical step is to control the quality (QC) of the input CITE-Seq data. For QC of CITE-Seq data, previous studies performed a limited set of analyses, and there was no standalone method. To develop a desirable standalone method for CITE-Seq QC, we recognize the following three limitations in the current CITE-Seq studies. First, some studies performed QC only at the RNA level, e.g., in terms of either transcriptome library size (Butler et al., 2018; Stuart et al., 2021), transcriptomic technical artifacts such as RNA contamination (Hong et al., 2022), or likely empty droplets or ambient RNAs (Grob et al., 2023; Subramanian et al., 2022). However, since CITE-Seq assays both RNA and cell surface protein data, CITE-Seq QC must assess not only individual RNA quality but also the quality of protein data and their interactions with RNA data. Specifically, i) the individual protein and RNA data quality must be controlled, respectively, to faithfully identify cell types with certain surface proteins and capture the cells’ molecular profiles, and ii) the relationship between the RNAs and the proteins must be investigated since, if certain cells express a specific gene that is readily translated and transported to the surface, the surface protein abundance level is expected to be correlated with the gene expression in the cells. Second, while a small number of other studies used surface protein information for QC, they examined only a subset of the assayed surface proteins as they were interested in particular cell types marked by the surface proteins. For example, one study examined 7 protein markers (CD3, CD4, CD8, CD14, CD16, CD19, and CD56) out of 188 available markers in the data to differentiate five cell types (B cells, CD4 T cells, CD8 T cells, classical monocytes, and natural killer) (Nettersheim et al., 2022), and another study examined four protein markers, out of 17 available markers, to differentiate four cell types (T cells, monocytes, B cells, and cytotoxic T lymphocytes) (Granja et al., 2019). However, to detect systematic errors that affect most assays in the data, it is important to examine the majority of RNAs and proteins rather than a small subset of them. Third, when the abovementioned studies demonstrated the relationship between genes and the corresponding proteins, they relied mostly on visual inspection of a dimensionality-reduced space (e.g., UMAP) for either the abundance level relationship between genes and proteins or their cell-type specificity. However, quantitative measures are needed to objectively assess the relationship between abundance levels and cell-type specificity. Quantitative measures can help further compare the data quality across various CITE-Seq datasets and make the QC analyses scalable.

In this study, we introduce CITESeQC, the first software package specifically designed to provide a comprehensive and interpretable set of quantitative metrics for assessing the quality of CITE-Seq data. Rather than performing direct filtering or removal of cells or features, CITESeQC serves as a diagnostic framework that guides users in making informed quality control (QC) decisions tailored to their dataset. CITESeQC supports multi-layered QC by offering seven modules for evaluating RNA or protein data individually and five additional modules for assessing cross-modality relationships, such as RNA–protein consistency. To ensure systematic coverage, these 12 modules collectively assess all genes and surface proteins in the dataset while flagging low-quality features using individual QC metrics. For quantitative evaluation, CITESeQC computes Shannon entropy to assess cell type-specific expression patterns and correlation coefficients to capture expected relationships between gene expression and protein abundance. Designed for broad usability, CITESeQC guides users through a streamlined process that generates a complete markdown report, including informative visualizations and interpretations, with minimal user intervention. This flexible, user-guided approach enables researchers to evaluate data quality in a nuanced and biologically informed manner—supporting both standardized workflows and exploratory analyses—without relying on rigid, pre-defined thresholds.

## Results

### CITESeQC quantifies various aspects of CITE-Seq quality

**FIGURE 1:**
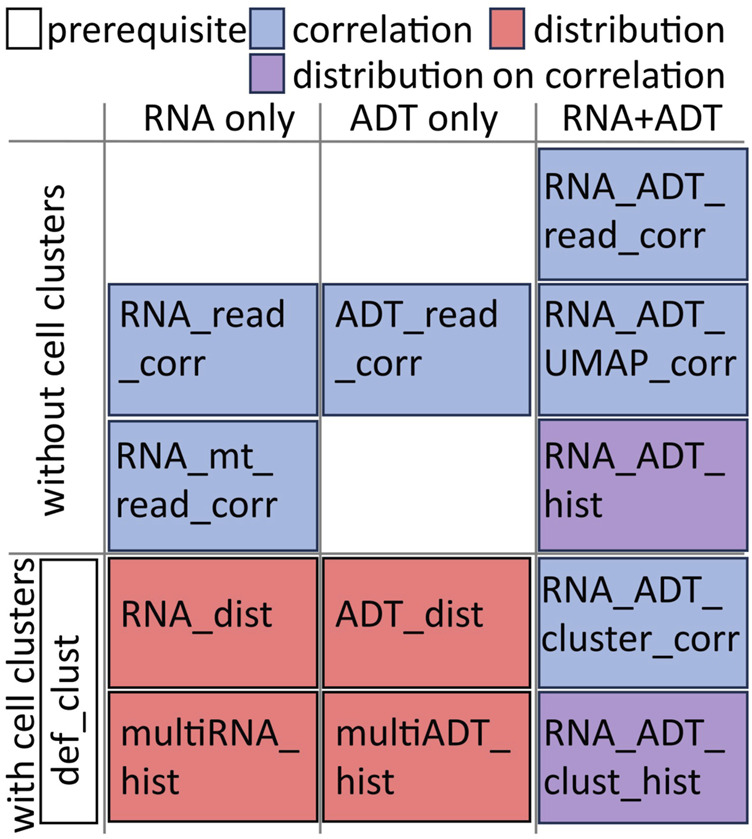
Illustrative categorization of the 12 R functions in CITESeQC. The additional def_clust() function needs to be run before running functions in “with cell clusters” category.

CITESeQC provides 12 R modules to assess the quality of RNAs, surface proteins, and their interactions in multiple aspects and one R module to define cell clusters or import cell cluster definitions (Figure 1). The modules also provide quantitative measures, wherever possible, to test particular hypotheses regarding the quality.

1. “RNA_read_corr()” produces a scatterplot correlating the number of molecules/genes with the number of genes identified in the transcriptome. Since the cutoffs for good-quality cells will be passed as the arguments to the function, users can modify them for their data. Default values are from the Seurat-guided clustering tutorial. Spearman’s correlation coefficient is calculated to allow users to test the hypothesis that the total number of genes increases with the number of detected genes in the transcriptome.
2. “ADT_read_corr()” produces a scatterplot correlating the number of detected ADTs with the total number of ADT molecules identified on the cell surfaces. Since the cutoffs identifying good-quality cells are annotated on the plot as passed as the arguments of the function, users can modify them for their data. Default values are from the Seurat-guided clustering tutorial. Spearman’s correlation coefficient is calculated to allow users to test the hypothesis that the total number of ADT molecules increases with the number of detected ADTs on the cell surface.
3. “RNA_mt_read_corr()” produces a scatterplot correlating the number of genes identified in the transcriptome with the percentage of the mitochondrial genes. Spearman’s correlation coefficient is calculated to allow users to test the hypothesis that the mitochondrial percentage remains constant regardless of the number of identified molecules.
4. “def_clust()” either defines the cell clusters based on the input gene expression matrix or imports the definition. To define the cell clusters, it employs the Seurat package with the input clustering resolution. For each cell cluster, whether defined internally or imported, this function identifies marker genes for later use.
5. “RNA_dist()” visualizes the specificity of the input gene expression across the cell clusters defined or imported using def_clust(). For quantification and comparison, it calculates Shannon entropy on the expression distribution across clusters, which is defined as follows: $H_{normalized}=-\frac{1}{\log_{2}\left(N\right)}\sum_{i=1}^{n}p_{i}\log_{2}\left(p_{i}\right)$ , where *N* is the number of clusters (size of the alphabet). A lower value in Shannon entropy represents a more specific expression of the gene across the clusters.
6. “multiRNA_hist()” is a histogram of Shannon entropy values of the marker genes identified in def_clust(). The histogram displays the specificity of marker genes across clusters. Users can modify the number of marker genes. A histogram peak at high entropy values suggests that the marker genes lack specificity.
7. “ADT_dist()” visualizes the specificity of the input ADT abundance across the cell clusters. Specifically, it calculates normalized Shannon entropy on the expression distribution across clusters. Note that the clusters were defined based on gene expression unless provided by the users.
8. “multiADT_hist()” is a histogram of normalized Shannon entropy values of all ADTs identified for the cell clusters. The histogram displays the specificity of ADT markers across clusters. Note that the clusters were defined based on gene expression unless provided by the users. A histogram peak at high entropy values suggests that the marker genes lack specificity.
9. “RNA_ADT_read_corr()” produces a scatterplot showing the correlation between the number of assayed genes in the transcriptome and the number of assayed cell surface proteins across the cells. Spearman’s correlation coefficient is calculated to allow users to test the hypothesis that the number of assayed proteins increases with the number of assayed genes.
10. “RNA_ADT_UMAP_corr()” produces pairs of UMAP plots and a scatterplot. Each UMAP plot pair is drawn for the abundance of the input ADT and the corresponding gene expression, respectively. The scatterplot plots the abundance of ADTs and the expression of the RNAs of the input gene.
11. “RNA_ADT_cluster_corr()” is a set of scatterplots, each drawn for each cell cluster, showing the correlation between input ADT abundance and the corresponding gene expression for the cluster.
12. “RNA_ADT_hist()” is a histogram of the correlation coefficients in all pairs of ADTs and the corresponding genes in expression.
13. “RNA_ADT_cluster_hist()” is a set of histograms, each showing the distribution of the correlation coefficients in all pairs of ADTs and the corresponding genes for each cell cluster.

### CITESeQC interpretation of diagnostic quality metrics

**FIGURE 2:**
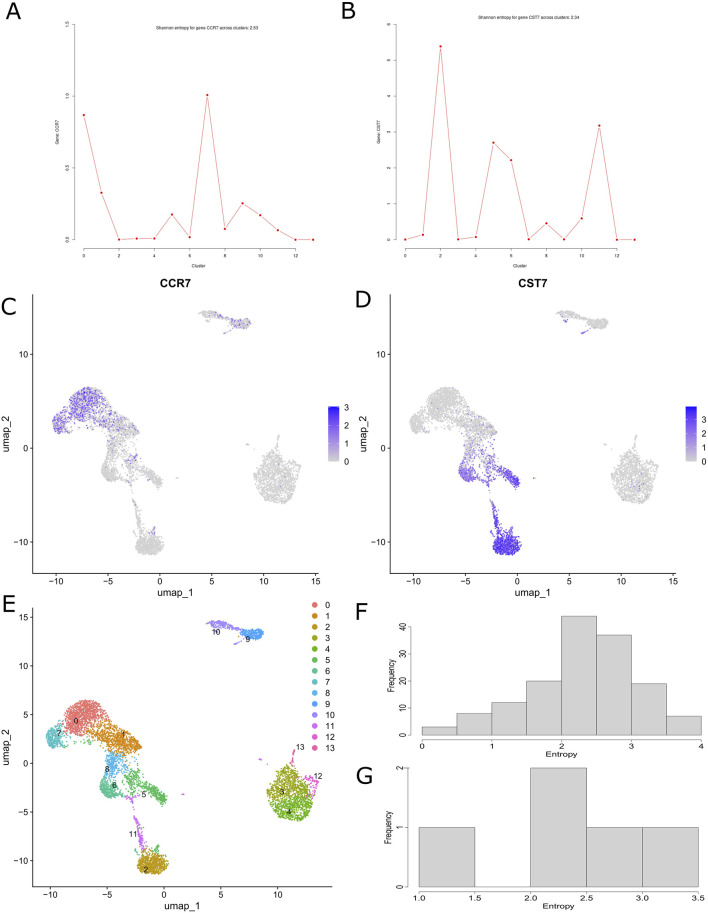
CITESeQC functions showing and quantifying relative abundance distribution of **(A)** CCR7 RNA and **(B)** CST7 in the example PBMC dataset. The amount of uncertainty in the probability distribution is measured by Shannon entropy. UMAP showing specificity across cell clusters for **(C)** CCR7 and **(D)** CST7. **(E)** UMAP showing the cell cluster definition in the PBMC dataset. CITESeQC functions showing the distribution of the Shannon entropy values of **(F)** the top 10 marker genes from each cluster and **(G)** all surface markers across the clusters defined in def_clust() on the example PBMC dataset.

We demonstrate the applicability of CITESeQC using two example CITE-Seq datasets from healthy donors. The first comprises peripheral blood mononuclear cells (PBMCs), and the second comprises cord blood mononuclear cells (CBMCs). On the datasets, three functions beginning with either “RNA” or “ADT” and ending with “read_corr” inspect the correlation between the total number of reads and those aligned with RNAs or proteins across cells, enabling users to test whether the alignment process contributes to the quality. CITESeQC calculates Spearman’s correlation coefficient and a permutation-based *p*-value as quantitative measures. Our analysis of PBMC and CBMC datasets (Supplementary Figures S1A–C, 2A–C) confirms that a valid alignment should yield a positive correlation. The functions RNA_dist() and ADT_dist() compute the distribution of a single marker gene or surface protein across cell clusters using Shannon entropy to quantify target specificity. To illustrate their utility, we examined CCR7 and CST7 in PBMCs—canonical markers for naïve T cells and cytotoxic lymphocytes, respectively (Figures 2A,B). Although both are recognized markers, Seurat’s built-in module lacks the resolution to differentiate their relative specificity across clusters (Figures 2C–E). In contrast, our entropy-based quantification provides a clear, interpretable measure of specificity. For example, CCR7 is less specific than CST7 (with entropy values of 2.53 and 2.34, respectively), enabling researchers to prioritize CST7 over CCR7 for downstream analyses, such as cell-type annotation, differential expression, and experimental validation. This added layer of interpretability represents a key advantage over existing methods. We also showed the specificity of CCR7 in CBMC and CD14 ADT in PBMC and CBMC (S. Figures 2D–F). CD14 also shows strong specificity across PBMC and CBMC cell clusters as it is robustly expressed in classical and intermediate monocytes, with Shannon entropy values of 2.39 and 3.83, respectively. “multiRNA_hist()” and “multiADT_hist()” visualize the distribution of Shannon entropy values for marker genes and surface proteins, respectively. In our analysis, we used the top 10 marker genes for each cluster and all surface proteins identified in PBMC and CBMC (Figures 2F,G,S; Figures 2G,H). In addition, three functions beginning with “RNA_ADT” and ending with “corr” allow practitioners to quantify the correlation between RNAs and surface proteins. Our analysis of CD14 on PBMC and CCR7 on CBMC (Supplementary Figures S1D–G, 3, 4, 5) visually demonstrates their specificity across cell clusters on UMAP and using correlation. Finally, two functions beginning with “RNA_ADT” and ending with “hist” visualize the distribution of the correlation either across all clusters or for each cluster. Running the functions on CCR7 and ADT14 shows cluster-specific behavior of the markers (Supplementary Figures S6, 7). Before running functions that require cell cluster definitions (e.g., RNA_dist()), def_clust() should be called to either define or import them.

### Systematic evaluation of CITESeQC’s sensitivity to technical noise in CITE-Seq data

**FIGURE 3:**
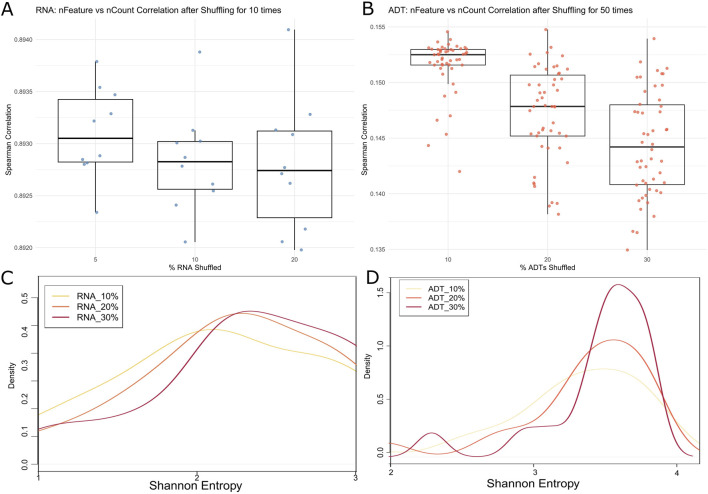
Spearman’s correlation of 10% randomly selected cells in the PBMC dataset estimated between nFeature and nCount of **(A)** RNAs after 5%, 10%, and 20% shuffling and **(B)** all ADTs after 10%, 20%, and 30% shuffling. Shannon entropy density plot of marker genes in 10% randomly selected cells of the PBMC dataset estimated across defined clusters of **(C)** RNAs after 10%, 20%, and 30% shuffling and **(D)** all ADTs after 10%, 20%, and 30% shuffling.

To show how CITESeQC detects systemic errors, we performed two controlled noise-injection experiments using 10% of the cells randomly selected in the PBMC dataset. First, to simulate noise introduced by systemic disruptions in feature-count relationships, we shuffled expression values for 5%, 10%, and 20% of RNA features and 10%, 20%, and 30% of ADT features. We selected higher percentages for ADT data to ensure a noticeable effect despite its smaller feature set (33,538 RNAs vs. 17 ADTs). For RNA, each condition was repeated 10 times; for ADT, 50 times for statistical significance and computational efficiency. To quantify the noise effect, CITESeQC calculates Spearman’s correlation between nFeature (the number of unique genes or proteins detected in a cell) and nCount (total count per cell). In high-quality data, these metrics are expected to show a strong positive correlation—cells with more detected features tend to have higher total counts. Our shuffling strategy is to preserve cell-level relationships while disrupting the gene- or protein-level relationships. In the results, we observed a consistent decrease in correlation values with increasing levels of noise for both RNA and ADT (Figures 3A,B). The RNA modality showed a wider dynamic range of degradation due to its larger number of features. These results confirm that CITESeQC’s correlation-based metrics are sensitive to global disruptions and can effectively capture systemic quality issues. Second, we evaluated how increasing randomness affects gene/protein specificity across clusters, a key step for downstream analyses. We randomly shuffled 10%, 20%, and 30% of RNA and ADT features, respectively, and defined clusters using the function def_clust(). For efficiency, we selected 10,000 RNA features by ranking genes according to the standard deviation of their expression across cells and retaining those with the highest variability. Using the defined clusters, we ran multiRNA_hist() and multiADT_hist() functions to calculate the Shannon entropy across all shuffled features. In high-quality data, markers with specificity should show low entropy. As we increased the level of noise, the entropy values exhibited a systematic increase, with the overall distribution shifting toward higher values (i.e., rightward shift). For RNA features, we observed significant shifts in Shannon entropy from 10% to 20% and from 20% to 30% (*p*-value: 0.04 and 0.05, respectively, Figure 3C), suggesting a loss of cluster-specific expression patterns. A similar shift was found for ADT features, although it was not significant (*p*-value: 0.2 in both 10%–20% and 20%–30%, Figure 3D), potentially due to the limited number of measured ADTs (n = 17). These findings demonstrate that entropy-based metrics in CITESeQC effectively capture the erosion of biological signal due to random noise. Together, both experiments validate the sensitivity of CITESeQC to detect quality issues at multiple levels—global structure and cluster specificity—making it a valuable tool for CITE-Seq data QC across applications and platforms.

### CITESeQC facilitates marker specificity analysis

To demonstrate how CITESeQC’s quantitative measures can improve downstream biological analysis, we systematically determined a Shannon entropy cutoff to assess the specificity of marker genes. Specifically, we focused on defining an empirical threshold that distinguishes truly cluster-specific markers from background, non-specific genes. To establish this threshold, we first randomly selected 1,000 expressed RNAs (>5 in average expression) that were not differentially expressed across any clusters in the PBMC dataset to serve as a negative control. We then calculated the Shannon entropy of these non-marker genes across pre-defined clusters. Because these genes are expected to be broadly and non-specifically expressed, their entropy distribution reflects a null distribution of non-specific expression. We defined the marker specificity cutoff as the 5th percentile of this distribution (i.e., the left tail), identifying entropy values below this threshold as statistically specific. We then applied this empirical cutoff to evaluate the top 10, 20, and 30 RNA markers (ranked by differential expression p-value) identified in our analysis (Supplementary Figure S8). Although the set with more RNA markers exhibits heterogeneous distribution of entropy values, the cutoff clearly distinguishes significantly specific markers from non-specific markers. In PBMCs, for example, entropy values below 1.45 were deemed specific, with 26 (20%), 39(16%), and 41 (12%) of the top 10, 20, and 30 markers, respectively, meeting this criterion (Supplementary Table S1). In CBMCs, where the cutoff was 0.75, similar trends were observed. This analysis quantitatively validates which markers are truly specific to each cluster. By selecting cluster-specific markers based on CITESeQC entropy-based specificity, users can enhance the biological interpretability and clinical utility of single-cell data analyses. This is particularly important because high-specificity markers are essential for robust cell type classification, biomarker discovery, therapeutic targeting, and ensuring reproducibility across datasets.

## Discussion

The CITESeQC package is the first software package that assesses the quality of CITE-Seq data in terms of the individual RNAs, surface proteins, and their interactions. For quantitative evaluation, CITESeQC computes Shannon entropy and RNA–ADT correlation coefficients—two biologically informed metrics. Although entropy itself is designed to quantify expression distribution and is not a direct indicator of technical quality, it becomes informative about data quality when applied to marker genes or proteins. In high-quality CITE-Seq data, well-established cell type markers—such as CD3 for T cells or CD19 for B cells—should exhibit low entropy, with expression localized to the expected clusters. If these canonical markers instead show unexpectedly high entropy—that is, broadly or randomly distributed expression—it may suggest technical issues such as ambient RNA contamination that causes marker expression to bleed into unrelated clusters, poor clustering resolution that reflects insufficient transcriptomic signal, or antibody non-specificity or background staining in the ADT layer. Similarly, for a subset of well-characterized, high-expression surface markers, a moderate to strong positive correlation between mRNA and protein levels is expected in biologically consistent and technically sound CITE-Seq data. When known concordant markers exhibit unexpectedly low or erratic correlations, it can suggest technical artifacts such as antibody dropout or mislabeling, droplet barcoding or ambient tag misassignment, or batch effects or sample degradation. CITESeQC does not use these metrics to impose strict thresholds or automatically discard features; instead, it provides them as diagnostic tools to allow users to distinguish between meaningful biological heterogeneity and technical noise. Altogether, we provide a comprehensive set of computational QC measures for CITE-Seq data that assess and quantify various aspects of data quality at both individual RNA and protein levels and in their interactions.

To determine the quality of a CITE-Seq dataset using the quantitative measures provided by CITESeQC, the next step is to determine appropriate cutoff values for each measure. However, establishing some cutoff values is not straightforward. For example, measures correlating RNAs with their corresponding surface proteins depend not only on data quality but also on the translation efficiency of the RNAs. Even for datasets of same quality, translation efficiency can vary across biological contexts due to post-transcriptional regulatory processes such as alternative polyadenylation and competing endogenous RNAs (Fan, et al., 2020; Kim, et al., 2020; Park, et al., 2018). Thus, to assess quality using correlation measures, we recommend comparing the values with those from other CITE-Seq datasets for which users have prior knowledge of data quality. In the future, to perform QC analysis without reference datasets, we plan to collect multiple CITE-Seq datasets of both high and low quality and determine cutoff values directly from the data.

## Methods

### CITESeQC in user-friendly R markdown

CITESeQC (version 0.9.1) is an R package with minimal prerequisites and is publicly available at https://github.com/sunjie001130/CITESeQC. It employs the baseline R packages—graphics, stats, and utils—making it and easy for users to install. Both the source code and tutorial with example datasets are available to download. The tool can be used in an R script or R Markdown file. The advantage of this design is that it can allow the integration of code, visualizations, and explanations in a single document, which facilitates reproducibility and documentation of data analysis workflows. Additionally, R markdown files do not require familiarity with command-line syntax, like many Linux environment-based software programs.

### Experiment data

PBMCs, which have a single round nucleus, include lymphocytes (T cells, B cells, and NK cells) and monocytes isolated from peripheral blood. We downloaded the dataset from https://www.10xgenomics.com/, and CBMCs are derived from umbilical cord blood. They include hematopoietic stem/progenitor cells and immune cells that are more naive than adult PBMCs, making them valuable for studying immune development. We downloaded the dataset from https://www.ncbi.nlm.nih.gov/geo/query/acc.cgi?acc=GSE100866.

## Data Availability

The original contributions presented in the study are included in the article/Supplementary Material; further inquiries can be directed to the corresponding author.

## Supplementary material

SUPPLEMENTARY FIGURE S1 CITESeQC functions based on correlation drawn for the example PBMC CITE-Seq dataset. **(A)** The number of detected genes in each cell is plotted with the number of molecules. The cutoffs for cells of good quality are annotated on the plot between red lines. The correlation coefficient and *p*-value are estimated based on Spearman’s correlation. **(B)** The number of detected ADTs in each cell is plotted with the number of ADT molecules. The correlation coefficient and p-value are estimated based on Spearman’s correlation. **(C)** The number of molecules identified in the transcriptome is plotted with the percentage of the mitochondrial genes. The correlation trend can test whether the mitochondrial percentage remains constant regardless of the number of identified molecules. CITESeQC functions on the example PBMC dataset showing the distribution of **(D)** CD14 RNA and **(E)** ADTs for CD14 on the UMAP space, respectively. **(F)** Scatterplot plotting all the cells by the number of ADTs for CD14 and the expression level of RNA molecules of CD14. **(G)** Scatterplot plotting all the cells by the total number of all ADTs on the surface and all RNA molecules in the transcriptome.

SUPPLEMENTARY FIGURE S2 CITESeQC functions based on correlation drawn for the example CBMC CITE-Seq dataset. **(A)** The number of detected genes in each cell is plotted with the number of molecules. The cutoffs for cells of good quality are annotated on the plot between red lines. The correlation coefficient and *p*-value are estimated based on Spearman’s correlation. **(B)** The number of detected ADTs in each cell is plotted with the number of ADT molecules. The correlation coefficient and *p*-value are estimated based on Spearman’s correlation. **(C)** The number of molecules identified in the transcriptome is plotted with the percentage of the mitochondrial genes. The correlation trend can test whether the mitochondrial percentage remains constant regardless of the number of identified molecules. CITESeQC functions showing and quantifying relative abundance distribution of **(D)** CCR7 RNA, **(E)** ADT-CD14 in the example CBMC dataset, and **(F)** ADT-CD14 in the PBMC dataset. CITESeQC functions showing the distribution of the Shannon entropy values of **(G)** the top 10 marker genes from each cluster and (H) all surface markers across the clusters defined in def_clust() on the example CBMC dataset.

SUPPLEMENTARY FIGURE S3 Set of scatterplots **(A-O)** each drawn for each cell cluster in the PBMC dataset, showing the correlation between ADT-CD14 abundance and the corresponding gene CD14 expression.

SUPPLEMENTARY FIGURE S4 CITESeQC functions on the example CBMC dataset showing the distribution of **(A)** CCR7 RNA and **(B)** ADTs for CCR7 on the UMAP space, respectively. **(C)** Scatterplot plotting all the cells by the number of ADTs for CCR7 and the expression level of RNA molecules of CCR7. **(D)** Scatterplot plotting all the cells by the total number of all ADTs on the surface and all RNA molecules in the transcriptome.

SUPPLEMENTARY FIGURE S5 Set of scatterplots **(A-R)** each drawn for each cell cluster in the CBMC dataset, showing the correlation between ADT-CCR7 abundance and the corresponding gene CCR7 expression.

SUPPLEMENTARY FIGURE S6 Set of histograms for PBMC data, each showing the distribution of the correlation coefficients in all pairs of ADTs and the corresponding genes across all cell clusters **(A)** or for each cell cluster **(B-P)**.

SUPPLEMENTARY FIGURE S7 Set of histograms for CBMC data, each showing the distribution of the correlation coefficients in all pairs of ADTs and the corresponding genes for each cell cluster **(A-R)**.

SUPPLEMENTARY FIGURE S8 Shannon entropy density plot of marker genes across defined clusters of RNAs after 5%, 10%, and 20% shuffling in 10% randomly selected cells of the **(A)** PBMC and **(B)** CBMC datasets with negative control density generated from 1,000 expressed non-DE genes (gray shade).
