# Supplementary figures and images for: Quantitative measures to assess the quality of cellular indexing of transcriptomes and epitopes by sequencing data
Source: Front Bioinform. 2025 Sep 18;5:1630161. doi: 10.3389/fbinf.2025.1630161 (PMC12488637; doi:10.3389/fbinf.2025.1630161)

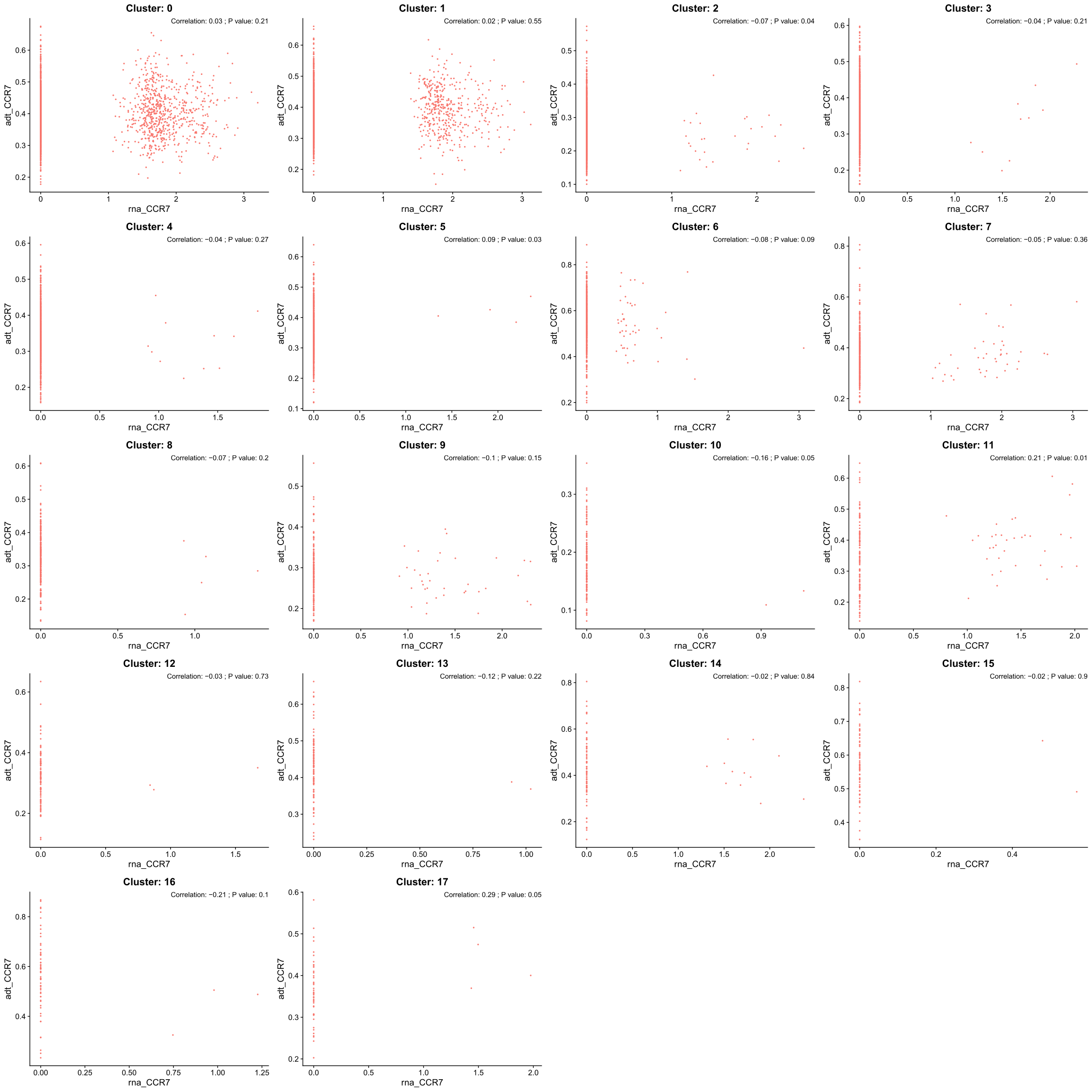

Supplement: Supplementary file 1 [file Image5.png]

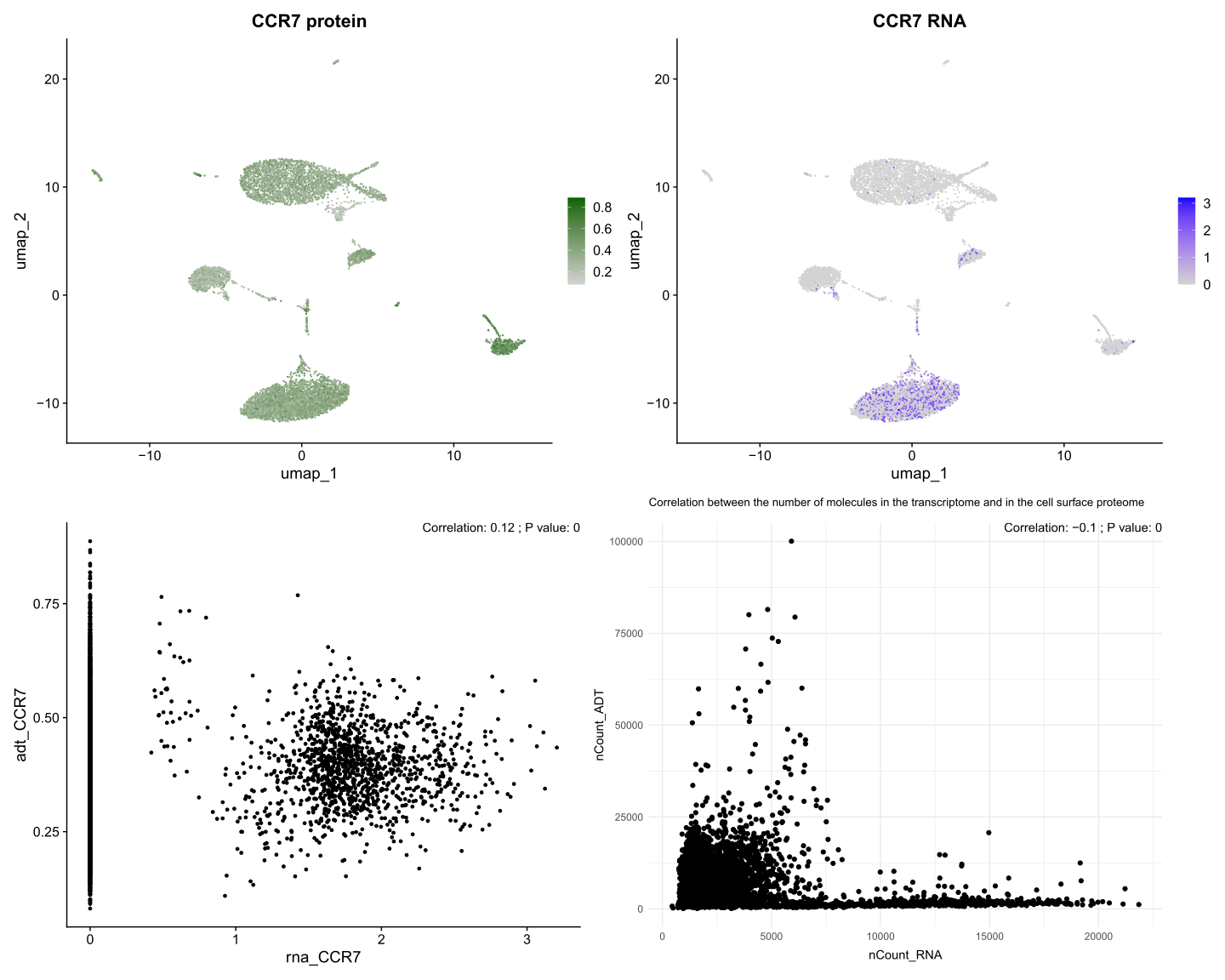

Supplement: Supplementary file 2 [file Image4.png]

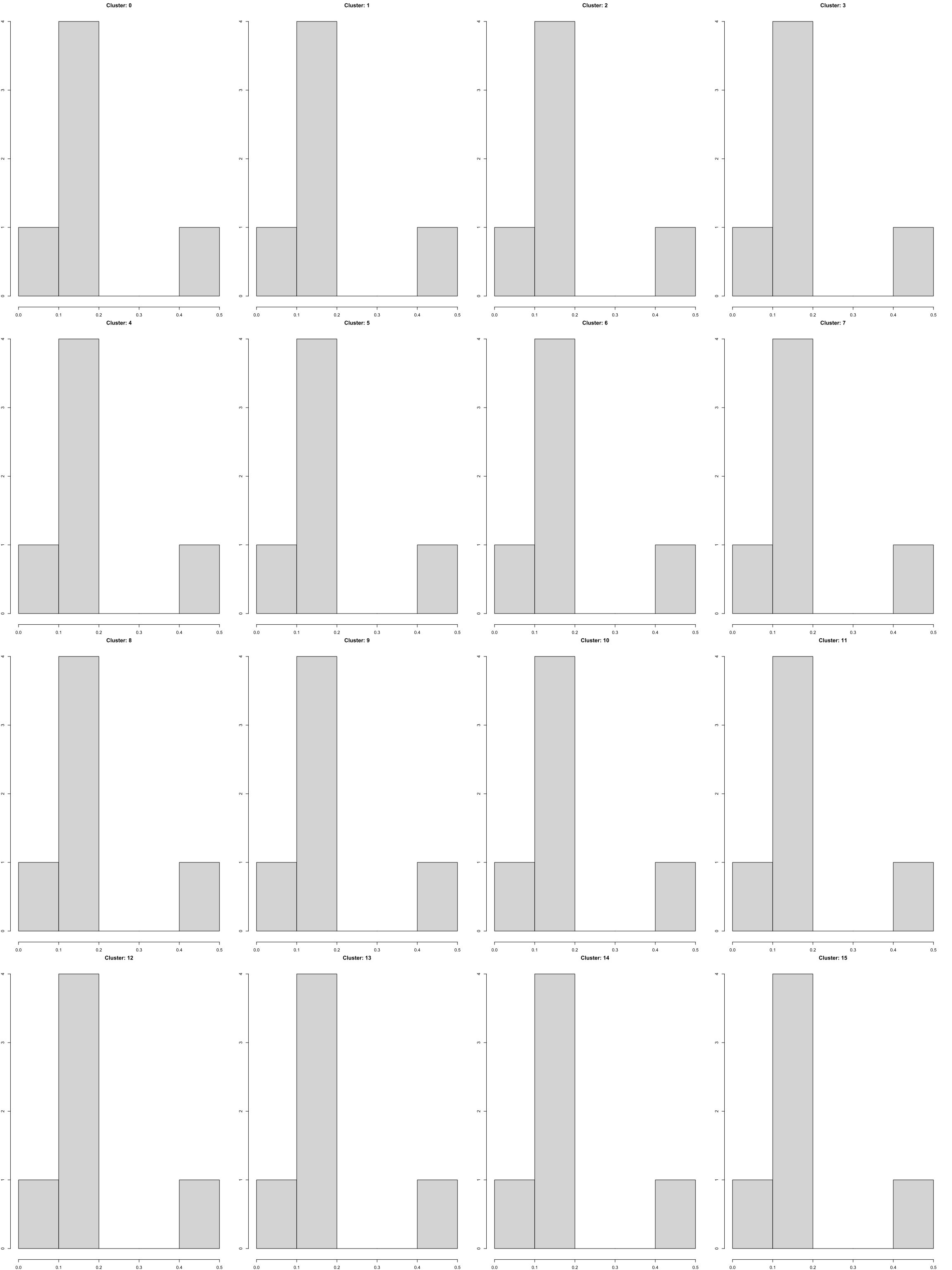

Supplement: Supplementary file 3 [file Image7.png]

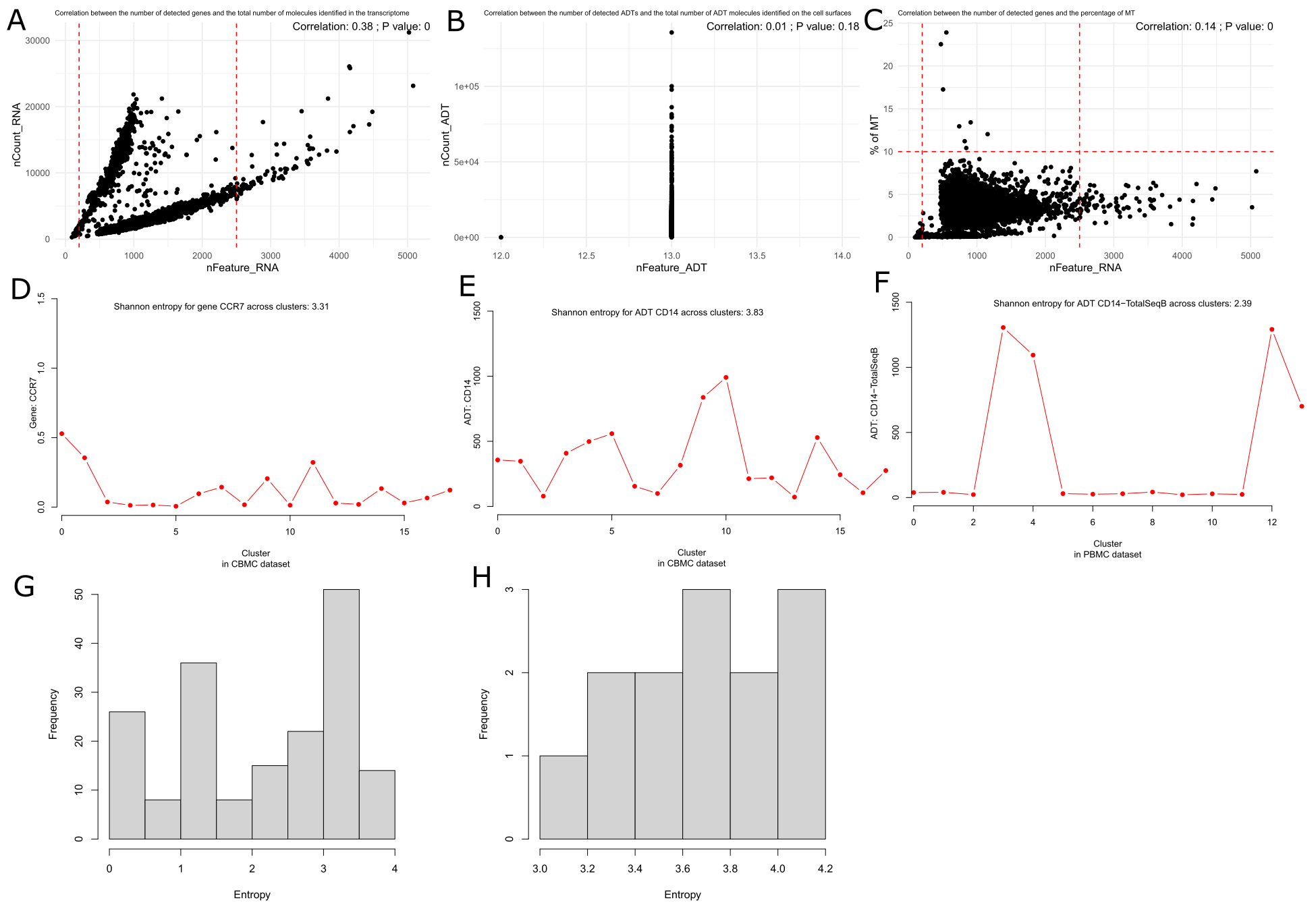

Supplement: Supplementary file 4 [file Image2.png]

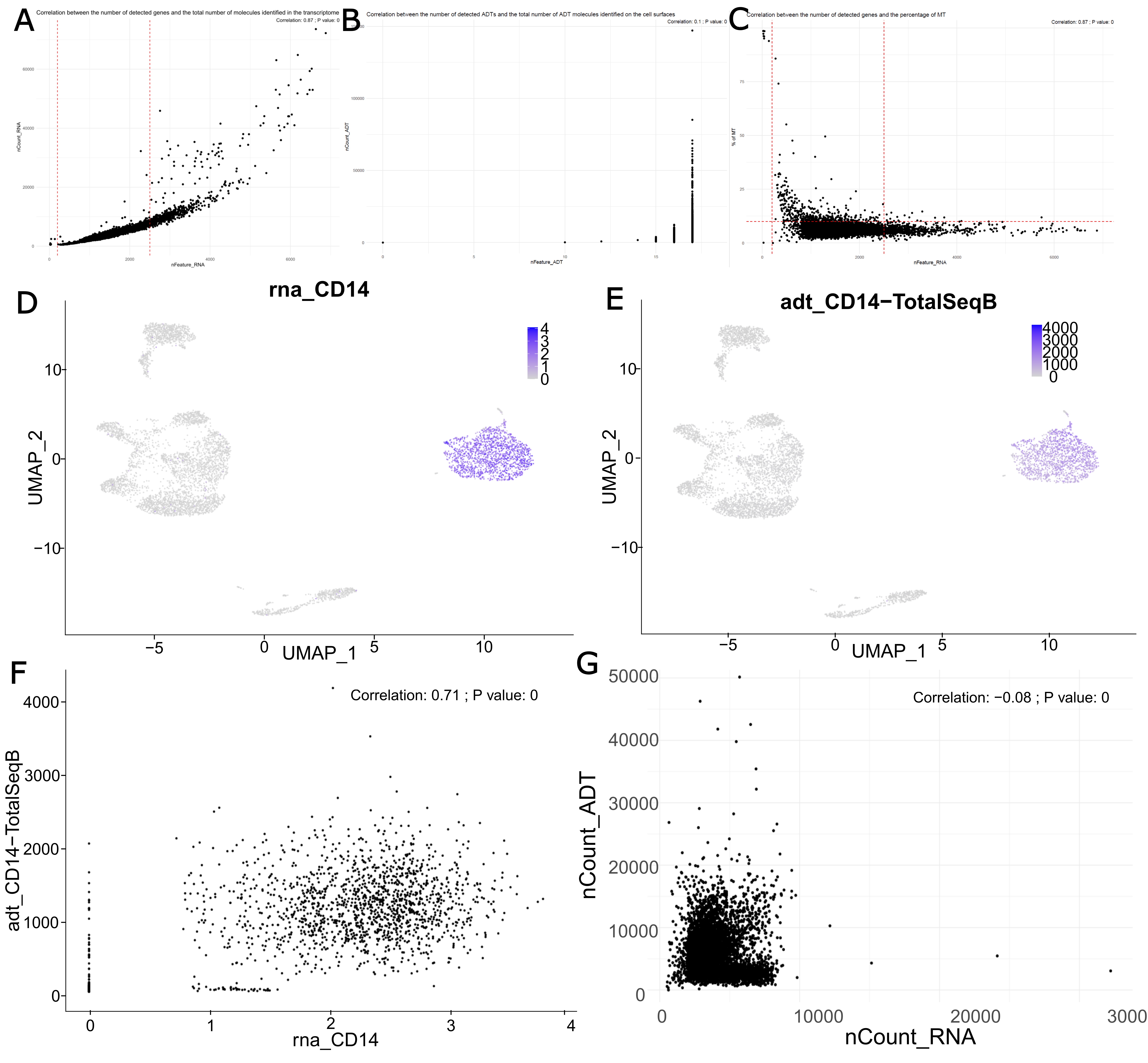

Supplement: Supplementary file 6 [file Image1.png]

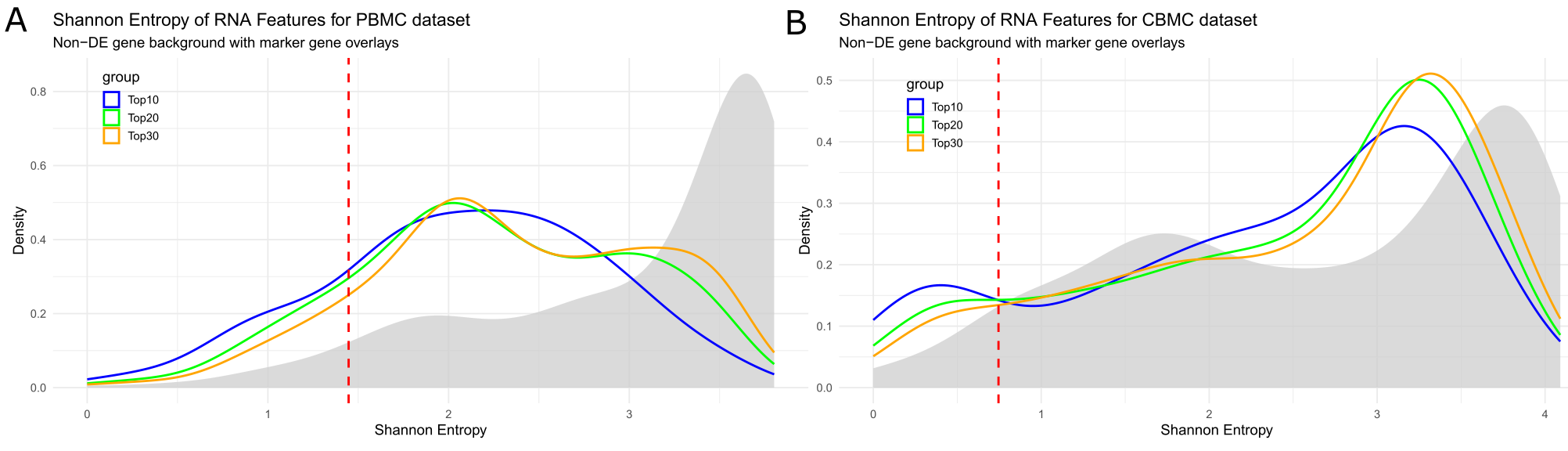

Supplement: Supplementary file 7 [file Image8.png]

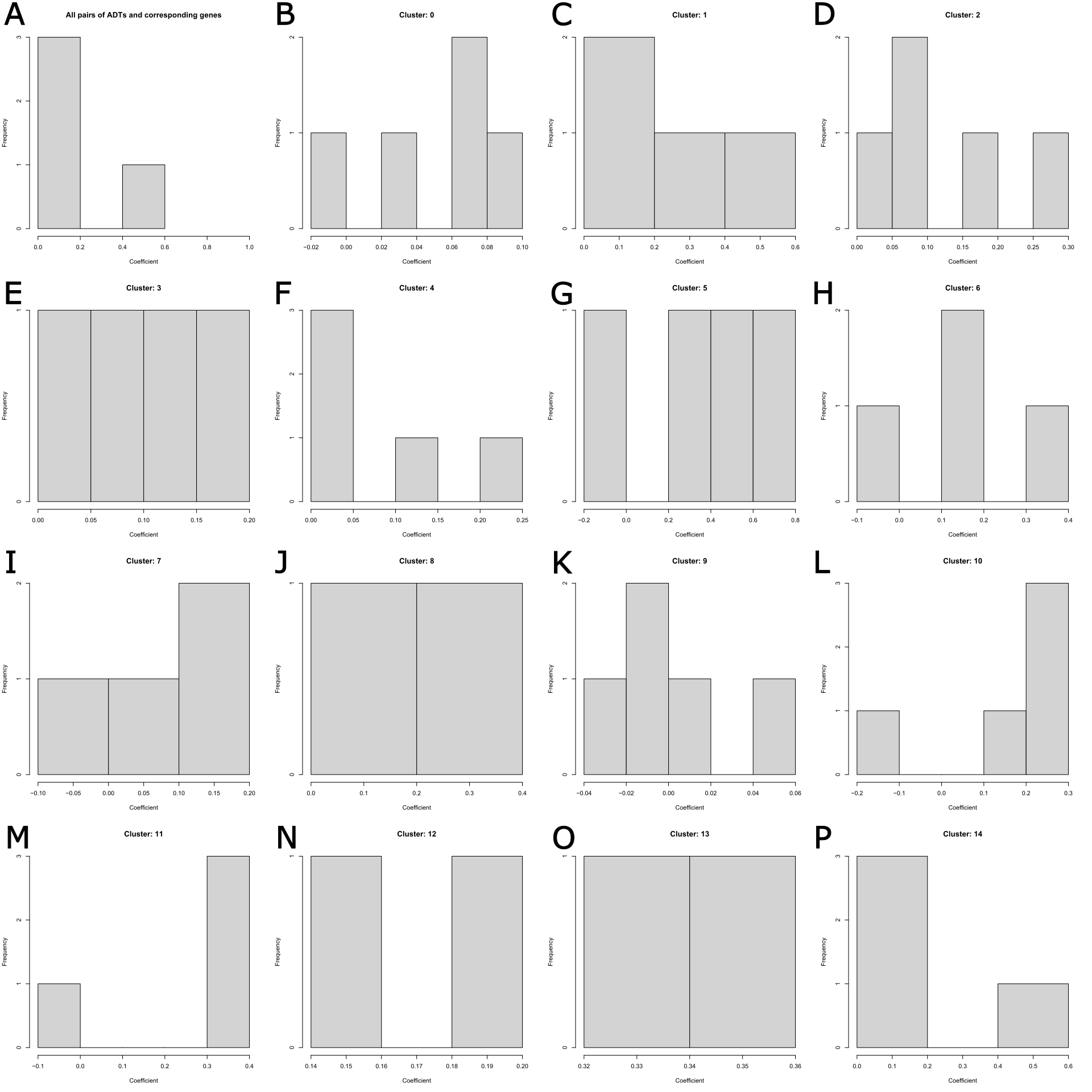

Supplement: Supplementary file 8 [file Image6.png]

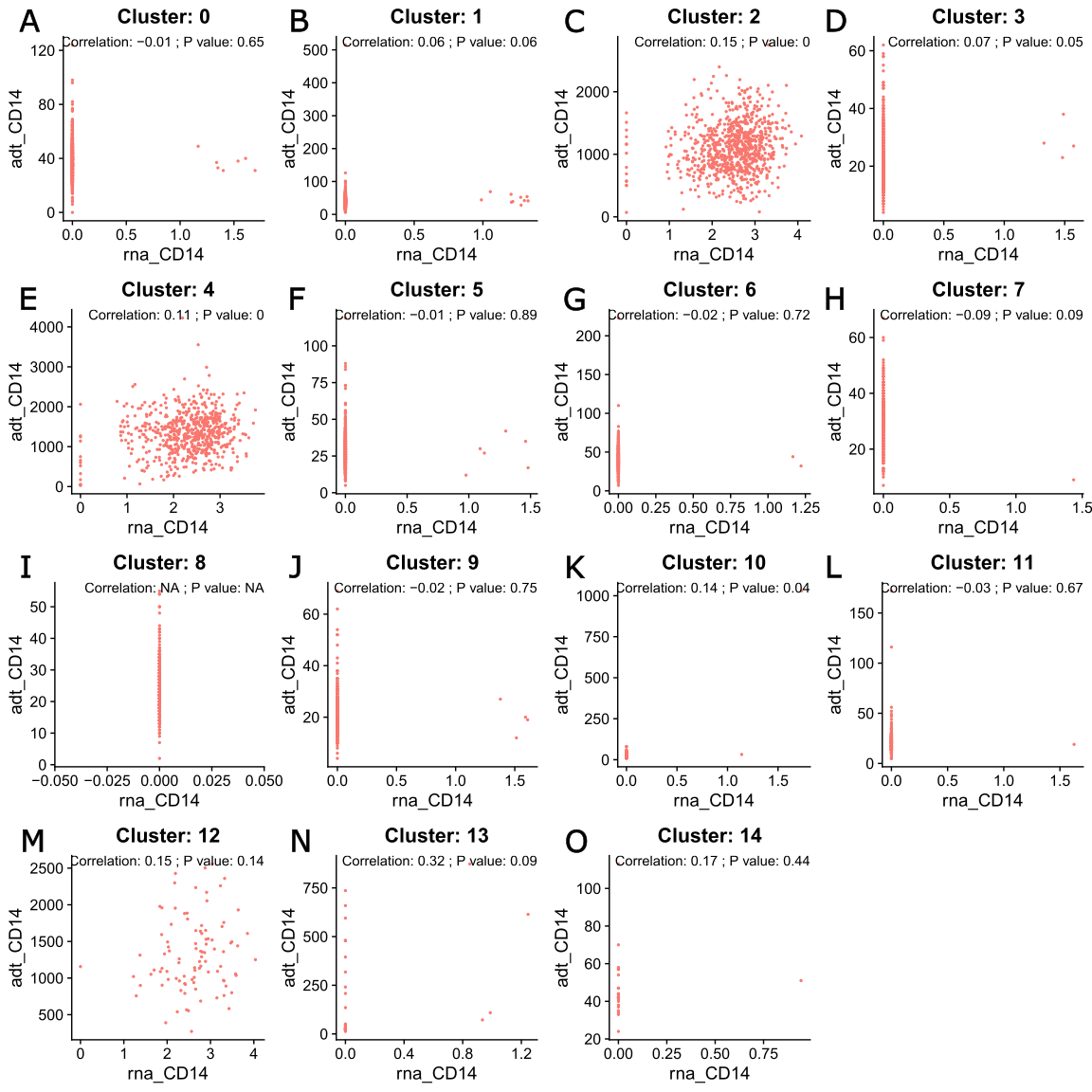

Supplement: Supplementary file 9 [file Image3.png]
